# Subsite-Specific Dietary Risk Factors for Colorectal Cancer: A Review of Cohort Studies

**DOI:** 10.1155/2013/703854

**Published:** 2013-03-12

**Authors:** Anette Hjartåker, Bjarte Aagnes, Trude Eid Robsahm, Hilde Langseth, Freddie Bray, Inger Kristin Larsen

**Affiliations:** ^1^Department of Nutrition, Institute of Basic Medical Sciences, University of Oslo, P.O. Box 1046 Blindern, 0317 Oslo, Norway; ^2^Department of Etiological Research, Cancer Registry of Norway, Institute of Population-Based Cancer Research, 0304 Oslo, Norway; ^3^Section of Cancer Information, International Agency for Research on Cancer, 69372 Lyon, France; ^4^Department of Registration, Cancer Registry of Norway, Institute of Population-Based Cancer Research, 0304 Oslo, Norway

## Abstract

*Objective*. A shift in the total incidence from left- to right-sided colon cancer has been reported and raises the question as to whether lifestyle risk factors are responsible for the changing subsite distribution of colon cancer. The present study provides a review of the subsite-specific risk estimates for the dietary components presently regarded as convincing or probable risk factors for colorectal cancer: red meat, processed meat, fiber, garlic, milk, calcium, and alcohol. *Methods*. Studies were identified by searching PubMed through October 8, 2012 and by reviewing reference lists. Thirty-two prospective cohort studies are included, and the estimates are compared by sex for each risk factor. *Results*. For alcohol, there seems to be a stronger association with rectal cancer than with colon cancer, and for meat a somewhat stronger association with distal colon and rectal cancer, relative to proximal colon cancer. For fiber, milk, and calcium, there were only minor differences in relative risk across subsites. No statement could be given regarding garlic. Overall, many of the subsite-specific risk estimates were nonsignificant, irrespective of exposure. *Conclusion*. For some dietary components the associations with risk of cancer of the rectum and distal colon appear stronger than for proximal colon, but not for all.

## 1. Introduction

Global estimates for 2008 indicate that colorectal cancer is the third most common cancer in the world [1]. Reports in several countries have described diverging incidence rates in colorectal cancer by subsite, including, in relative terms, an increasing proportion of proximal tumors [2–15], and thus a shift in absolute incidence from left- to right-sided colon cancers.

The reasons for this trend are not well understood; the subsites differ in physical function, artery supply, histology, and innervation, and they also derive from different segments in the primitive intestinal tract in the embryo [16]. The proximal colon originates from the midgut, whereas the distal colon and the rectum derivate originate from the hindgut. Comparisons have also shown that proximal colon tumors tend to have different molecular characteristics, with a higher proportion of microsatellite instability, and are more likely to have CpG island methylator phenotype and Ki-ras mutations than distal colon and rectal tumors [17].

It has been estimated that 45 percent of all colorectal cancer cases can be prevented in high-risk populations through modifications of diet, physical activity habits, and weight control [18]. According to the recent report from the World Cancer Research Fund/American Institute for Cancer Research (WCRF/AICR), there is convincing evidence that dietary fiber protects against colorectal cancer and that red and processed meat and alcohol (particularly in men) increase the risk of the disease [19]. Further, it is stated that garlic, milk, and calcium probably protect against colorectal cancer. No distinction is, however, made for the different subsites of the colorectum [19]. Also, although meta- [20–23] and pooled analyses [24–26] have provided quantitative synthesis for several of the dietary risk factors, little emphasis has been placed on subsite risks. 

Based on the biological differences in the colorectal segments and the reported differences in incidence, we may suggest differences across the segments in their association to lifestyle factors, such as diet. The aim of the present paper is to give an updated overview summarizing the etiological differences between the colorectal subsites with regard to the dietary factors considered to be convincing or probable risk factors for colorectal cancer. 

## 2. Material and Methods

The specific risk factors studied were red meat, processed meat, fiber, garlic, milk, calcium, and alcohol, selected given an *a priori* assessment of their importance in colorectal cancer etiology following the WCRF/AICR report in 2011 [19], and for which their modification could lead to a reduction in rates of colorectal cancer. The outcome was the risk of primary colorectal cancer according to subsite. 

**Figure 1:**
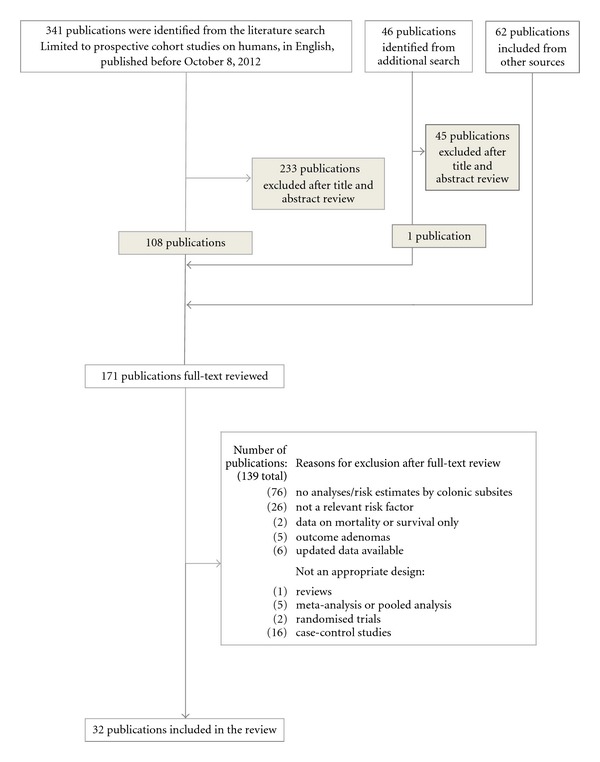
Flow diagram of the study selection.

A search for cohort studies published as original articles was conducted via a search of PubMed (http://www.ncbi.nlm.nih.gov), using a search strategy that combined the term “colorectal neoplasms” with the terms “risk factors” and “cohort study” with either the term “diet,” “nutrition,” or “alcohol.” The search was restricted to studies published or available online as of October 8, 2012, in the English language. A detailed description of the search strategy and the resulting papers retrieved is given in Table 1, and the procedure is described in Figure 1. A total of 341 articles were identified and reviewed according to title and abstract. The initial evaluation yielded 108 articles in the study database and underwent a second evaluation based on full-text review. A similar PubMed search for case-control studies nested within a cohort identified 46 articles, of which one underwent full-text review. In addition, further 62 articles were identified by scanning the reference lists of retrieved articles, reviews, meta-, and pooled analyses and underwent full-text review (Figure 1).

Studies were included if they provided risk estimates (and corresponding confidence intervals) for both proximal and distal colon cancer. Proximal colon (right sided) includes ceacum, ascending and transverse colon, while distal colon (left-sided) includes descending colon, sigmoid flexure, and sigmoideum. Some studies have not followed the above-mentioned classification [27–29], and this is specified in Table 2. Further, to be included, the cohorts had to be either population based, registry based, or obtained from censuses. Studies on specific subpopulations (e.g., hospital-based cohorts) were not included nor were studies examining second cancers, metastasis, survival, or mortality. Following the full-text evaluation, 139 articles were excluded for the reasons indicated in Figure 1, and 32 articles were included in the review. Of these, seven gave data on (red) meat [30–36], five on processed meat [31–35], eight on fiber [37–44], one on garlic [37], three on milk [45–47], seven on calcium [28, 45, 47–51], and ten on alcohol [27, 29, 36, 52–58]. 

**Figure 2:**
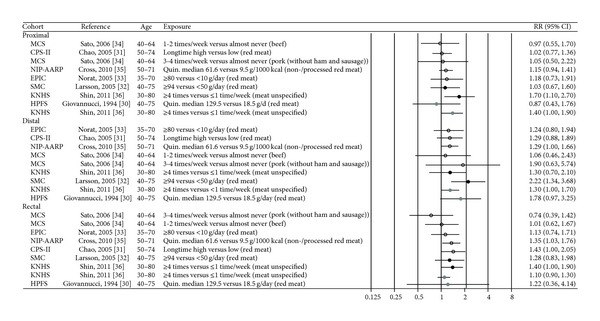
Estimates of relative risk with 95% CI for the highest versus the lowest exposure categories of red meat. (The results are stratified on sex. Open circles: both gender combined. Closed black circles: females. Closed grey circles: men. All estimates are sorted from the lowest to the highest by subsite and sex.)

**Figure 3:**
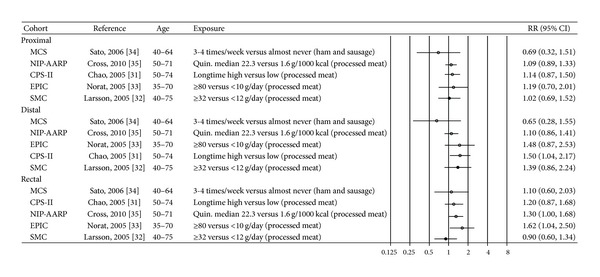
Estimates of relative risk with 95% CI for the highest versus the lowest exposure categories of processed meat. (The results are stratified on sex. Open circles: both gender combined. Closed black circles: females. All estimates are sorted from the lowest to the highest by subsite and sex.)

**Figure 4:**
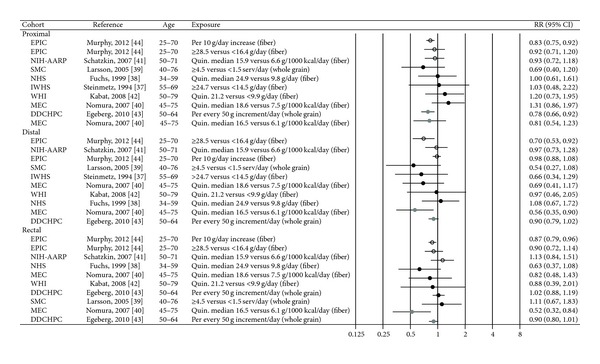
Estimates of relative risk with 95% CI for the highest versus the lowest exposure categories of fiber and whole grain. (The results are stratified on sex. Open circles: both gender combined. Closed black circles: females. Closed grey circles: men. All estimates are sorted from the lowest to the highest by subsite and sex.)

**Figure 5:**
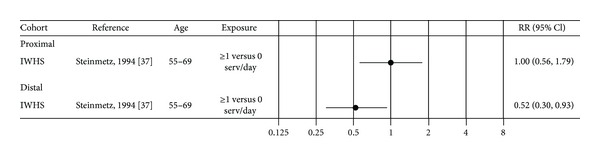
Estimates of relative risk with 95% CI for the highest versus the lowest exposure categories of garlic. (Closed black circles: females.)

**Figure 6:**
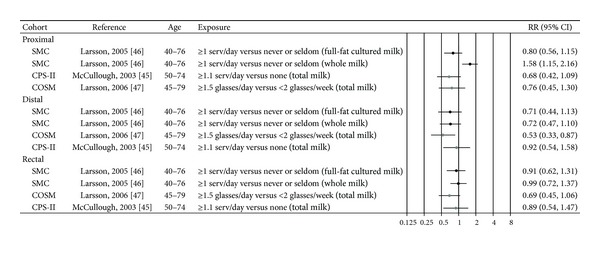
Estimates of relative risk with 95% CI for the highest versus the lowest exposure categories of milk. (The results are stratified on sex. Closed black circles: females. Closed grey circles: men. All estimates are sorted from the the lowest to the highest by subsite and sex.)

**Figure 7:**
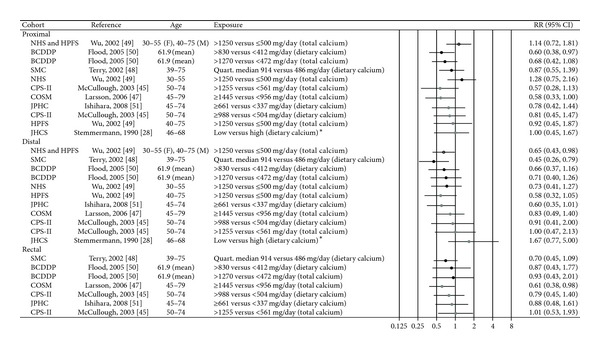
Estimates of relative risk with 95% CI for the highest versus the lowest exposure categories of calcium. (The results are stratified on sex. Open circles: both gender combined. Closed black circles: females. Closed grey circles: men. All estimates are sorted from the lowest to the highest by subsite and sex, except Stemmermann et al. [28].)

**Figure 8:**
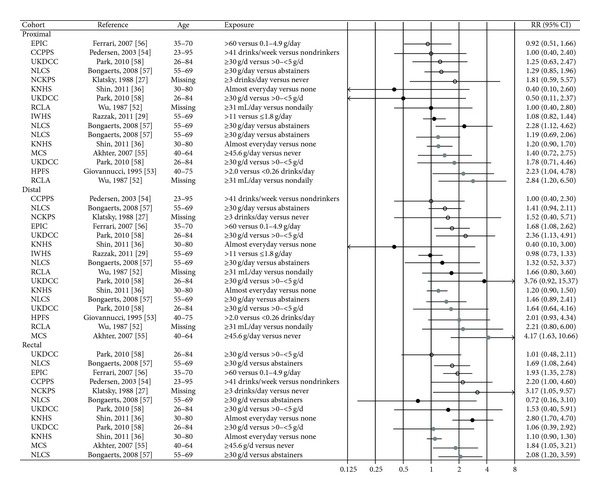
Estimates of relative risk with 95% CI for the highest versus the lowest exposure categories of alcohol. (The results are stratified on sex. Open circles: both gender combined. Closed black circles: females. Closed grey circles: men. All estimates are sorted from the lowest to the highest by subsite and sex.)

The number adds up to more than 32 as several papers give data for more than one risk factor. If several papers on the same risk factor were published for a given cohort, all data was retrieved from the most recent paper. The relative risk (RR), hazard rate ratio (HRR), hazard ratio (HR), incidence rate ratio (IRR) or odds ratio (OR), and corresponding 95% confidence intervals (95% CI) for each risk factor are presented (as RR) in Figures 2–8, sorted in ascending order of magnitude by sex. For one study the risk estimates were tabulated according to the lowest versus highest exposure category in the original paper [28] and are presented as the inverse of the value in the corresponding figure (Figure 7). 

Meta-analyses were not performed as a consequence of the heterogeneity in factors such as exposure measurement, the categorization of risk factor levels, and the confounders adjusted for in the studies [59].

## 3. Results

Overall, data from 21 cohorts with information on one or more of the risk factors were included in this review. Table 2 gives a detailed description of the studies, with information on cohort size, number of cases, sex, age distribution, follow-up time, and factors adjusted for in the risk analyses. The European Prospective Investigation into Cancer and Nutrition (EPIC) study consists of subcohorts from 10 European countries. The other cohorts were from the USA (10 cohorts), Sweden (2), Denmark (2), Japan (3), Korea (1), UK (1), and the Netherlands (1). The subsite-specific results (Figures 2–8) are described according to the individual risk factors, as presented in the following.

### 3.1. Red Meat

Seven cohort studies were included in the review, of which five reported on red meat [30–33, 35], one on beef and pork [34], and one on meat (not further specified) [36]. The five studies on red meat reported an increased risk of colorectal cancer with increasing intake [30–33, 35] (Table 2), although most of the risk estimates were not statistically different from unity. In Figure 2, there appears to be somewhat more consistency with the observation of an increased risk of cancers of the rectum and distal colon than there is for proximal colon cancer. This picture remains when restricting the evaluation to studies with adequate statistical power. In a Swedish study, women consuming 94 or more grams of red meat per day had an increased risk of distal colon cancer of 2.22 (95% CI 1.34–3.68) compared to women consuming less than 50 grams/day, and there was a significant trend of increasing risk with increasing consumption (*P*_trend_ < 0.001) [32]. A long-term high intake of red meat yielded an increased risk of rectal cancer of 43% (95% CI 1.00–2.05) relative to low intake in a US study [31], whereas in another US study there was a significant trend of increased risk of both proximal colon, distal colon and rectal cancer with increasing consumption of red meat (including both processed and nonprocessed red meat) (for all *P*_trend_ = 0.02) [35]. 

Neither beef consumption nor pork consumption was significantly associated with risk of cancer of any colorectal subsite in a Japanese study [34], while the frequency of meat consumption (not further specified) was positively associated with proximal and distal colon cancer in South Korean men, and with proximal colon and rectal cancer in South Korean women [36]. 

### 3.2. Processed Meat

Four studies have reported on processed meat [31–33, 35] and one has reported on ham and sausages [34] (Table 2). In the EPIC study, consumption of 80 grams or more of processed meat per day conferred a 62% (95% CI 1.04–2.50) increased risk of rectal cancer compared to an intake of less than 10 grams per day [33], and in a US study a high long-term intake of processed meat was associated with a 50% (95% CI 1.04–2.17) increased risk of distal colon cancer [31] (Figure 3). In a more recent American study, consuming 22.3 grams or more of processed meat per 1000 kcal increased the risk of rectal cancer with 30% (95% CI 1.00–1.68) compared to consuming 1.6 gram or less per 1000 kcal [35]. No significant associations were seen in Swedish [32] and Japanese studies [34]. 

### 3.3. Fiber

Eight cohort studies were included in the review; six have provided estimates for the risk of the colonic subsites in relation to fiber consumption [37, 40–42, 44] and two have provided estimates for the whole grain consumption [39, 43] (Table 2). As for fiber, most of the risk estimates were not statistically different from unity [37, 41, 42] (Figure 4). The EPIC study reported an inverse association between fiber intake and colorectal cancer with no strong evidence of different associations across the subsites: when fiber intake was analyzed as a categorical variable, a significant inverse association was seen for distal colon cancer only (*P*_trend_ = 0.02), whereas when fiber intake was analyzed as a continuous variable and corrected for measurement errors, significant inverse associations were seen for proximal colon (HR per 10 gram/day increase 0.83, 95% CI 0.75–0.92) and rectum cancer (HR per 10 gram day/increase 0.87, 95% CI 0.79–0.96), but not for distal colon cancer [44]. The Multiethnic Cohort observed a reduced risk for distal colon and rectal cancer among those in the highest compared to the lowest fiber intake group, but the reduction was only significant in men (RR 0.56, 95% CI 0.35–0.90 and RR 0.52, 95% CI 0.32–0.84, resp.) [40]. 

Two Scandinavian papers have reported on associations between whole grain consumption and cancer risk of colorectal subsites [39, 43]. In a Danish study, which is also included in the EPIC study, total consumption of whole grain products was associated with a significantly lower risk of proximal colon cancer and a borderline significantly lower risk of distal colon cancer and rectal cancer in men (IRR per each increment in intake of 50 gram per day 0.78, 95% CI 0.66–0.92 for proximal colon, 0.90, 95% CI 0.79–1.02 for distal colon, and 0.90, 95% CI 0.80–1.01 for rectum) but not in women (data not given) [43]. No significant associations between whole grain consumption and cancer of any of the colorectal subsites were seen in the Swedish Mammography Cohort [39] (Figure 4).

The width of the confidence intervals for the risk estimates on fiber and whole grain varies markedly between the included studies. However, whether all studies are included or those with the lowest power are excluded, there are rather minimal differences in relative risk across the subsites. 

### 3.4. Garlic

Only one study on garlic is included in the review. The Iowa Women's Health Study reported that having at least one serving per week of garlic was associated with a 48% reduced risk of distal colon cancer compared to zero servings of garlic (RR 0.52, 95% CI 0.30–0.93) [37] (Table 2, Figure 5). 

### 3.5. Milk

Three cohort studies were included in the review [45–47]. Two cohort studies have examined the relation between total consumption of milk and the risk of cancer in colorectal subsites [45, 47] (Table 2, Figure 6). No significant associations were seen for any of the colorectal subsites in a US study that combined both sexes (estimates not given for women) [45]. In a study of Swedish men, a significantly reduced risk for distal colon cancer was seen for those consuming 1.5 glasses or more of milk per day compared to those consuming less than two glasses per week (RR 0.53, 95% CI 0.33–0.87) [47]. 

In another Swedish study restricted to high fat dairy food and conducted among women only, a significant inverse trend was observed between consumption of full-fat cultured milk and risk of distal colon cancer (*P*_trend_ = 0.03), whereas a significant increased risk of proximal colon cancer was observed among women who consumed whole milk (1 or more servings per day compared to never/seldom consumers RR 1.58, 95% CI 1.15–2.16) [46]. 

### 3.6. Calcium

Seven papers from eight cohorts were included in the review [28, 45, 47–51] (Table 2, Figure 7). In the analyses from the Nurses' Health Study and the Health Professionals Follow-Up Study that studied colon cancer only, non-significant inverse associations were seen between total calcium and distal colon cancer for both men and women, and a pooled analysis of the two cohorts gave a significant inverse trend for distal colon cancer (*P*_trend_ = 0.01) [49]. Likewise, a significant inverse trend was reported between total calcium and distal colon cancer in the Swedish Mammography Cohort (*P*_trend_ = 0.02) [48], while in a US female cohort, a significant inverse trend was seen between dietary calcium intake and proximal colon cancer (*P*_trend_ = 0.01) [50]. No association was seen for total calcium. In a US study examining both sexes, total calcium intake was significantly inversely associated with proximal colon cancer among men (*P*_trend_ = 0.04), but not dietary calcium, and there were no significant associations seen for women (estimates not given) [45]. In a study of Swedish men, a significant inverse relation was found between total calcium and rectal cancer (*P*_trend_ = 0.02), whereas nonsignificant inverse associations were seen for cancer of the colon [47]. No significant associations between dietary calcium and cancer of any colorectal subsite were reported in a Japanese study either for men or for women (estimates not given for women) [51]. An earlier study of Hawaiian-Japanese men reported a significant inverse association between dietary calcium and sigmoid colon cancer (*P*_trend_ = 0.02) [28]. In addition to the eight cohorts included in this paper, an additional cohort study among American women states that their data provided little support for a protective effect of total calcium at either tumor subsite, but does not present risk estimates [60].

### 3.7. Alcohol

Ten articles [27, 29, 36, 52–58] were included in the review (Table 2). Three analyses for both sexes combined consistently showed a higher risk of rectal cancer with increasing alcohol consumption and no significant associations for any of the colon subsites [27, 54, 57]. In the EPIC study [56] an increased risk was reported both for rectal and distal colon cancer, whereas in the UK dietary cohort consortium (part of which is included in the EPIC study) [58] a significantly increased risk was found for distal colon cancer only (Figure 8). Several sex-specific analyses have been done. Among men, two older studies with limited statistical power reported that alcohol consumption was positively related to proximal colon cancer only [52, 53], whereas four more recent studies reported non-significant associations with proximal colon cancer [36, 55, 57, 58]. A study among Japanese men reported increased risk for distal colon cancer, but the confidence interval for the risk estimate was very wide (consumption of 45.6 grams alcohol or more per day compared to never drinkers HR 4.17, 95% CI 1.63–10.66) [55]. In terms of consumption of alcohol and rectum cancer among men, two of four studies have reported a positive association [55, 57]. 

For women, fewer significant associations have been reported. In the Iowa Women's Health Study, alcohol was not significantly associated with either proximal colon or distal colorectal cancer, nor did further separation into distal colon and rectal cancer reveal any significant associations (estimates not given) [29]. Furthermore, no significant associations were seen in an earlier study of American women [52] or in the UK dietary cohort consortium [58]. In The Netherlands Cohort Study, however, women consuming 30 grams or more of alcohol per day had HRR of proximal colon cancer of 2.28 (95% CI 1.12–4.62) compared to abstainers, whereas analyses for the other subsites were not sufficiently robust to draw any conclusions [57]. In a Korean study, women who frequently consumed alcohol or who consumed greater amounts of alcohol had a higher risk of rectal cancer [36]. 

## 4. Discussion 

This review provides an overall and updated synthesis of the results from cohort studies examining the association between dietary factors that are convincingly or probably related to the risk of colorectal cancer and subsite-specific colorectal cancer. Our study indicates that consumption of alcohol is more strongly related to the risk of rectal cancer than to colon cancer, also that meat consumption tends to be somewhat more strongly related to the risk of distal colon cancer and rectal cancer than proximal colon cancer, that there are only minor differences in relative risk for colorectal cancer across the major subsites for fiber, milk, and calcium, and that no statement can be given for garlic due to limited data. It should be noted that for all exposures the majority of the analyses showed nonsignificant associations with cancer risk, the exception being the positive association between alcohol consumption and rectal cancer. 

The pathway of colorectal cancer is complex. The subsite etiology is rather poorly understood, and the mechanism for the various dietary factors is likely to differ. Even for red and processed meat, both established risk factors for colorectal cancer, the underlying mechanisms are not well defined. One suggested mechanism for the somewhat stronger association for rectum and distal colon relative to proximal colon cancer relates to the enhanced endogenous formation of carcinogenic N-nitroso compounds with a high intake of meat [61]. The level of markers of N-nitroso compounds appears to be higher in tissue from distal colon and rectum than in that of the proximal colon [62]. This finding is in keeping with a previously meta-analyses which implicated processed meat consumption as a stronger risk factor for cancer occurrence at the distal colon relative to the proximal colon [22].

A suggested mechanism by which dietary fiber may decrease the risk of colorectal cancer is linked to the fermentation of fiber. The fermentation produces short-chain fatty acids and, in particular, acetic, propionic, and butyric acids. Butyrate is particularly of interest, as it has been shown to induce apoptosis and to be cytotoxic to both colorectal adenoma and carcinoma cells [63]. Studies on mice have shown that the concentration of butyrate is highest in the distal colon [64]. In humans, fiber is fermented in the proximal colon, and the total amount of short-chain fatty acids has been estimated to be considerably higher in the proximal site compared to the distal [65]. One study on proximal and distal colonocytes indicates, however, that butyrate is a more important source of energy for the distal, than for the proximal, colonic mucosa [66]. If so, this could be a relevant biological mechanism explaining any differences in risk between the sites. In addition, fiber may dilute the concentration of carcinogenic substances in the distal colon. Despite these findings, a pooled analysis on fiber reported no convincing differences in colorectal risk for the various anatomical subsites [25], in line with our own results published here. 

The reduced risk of colorectal cancer with increasing consumption of milk is likely to be at least partly mediated by calcium, which is thought to have a protective effect through its ability to bind bile acids, and its growth-restraining and differentiation- and apoptosis-inducing effect on colorectal cells [67]. No convincing site-specific associations regarding milk and calcium were seen in our review. A former pooled analysis reported an inverse association for milk limited to cancer of the distal colon and rectum [24], while the results from two meta-analysis on site-specific impact of calcium have been conflicting [20, 21].

The consumption of alcohol is associated with increasing risk of cancer in several organs in the digestive tract, including the colorectum [18]. Alcohol is not a carcinogen itself, but acts as a tumor promoter and possibly as a co-carcinogen. Alcohol also acts as a solvent and thus might increase the exposure to other carcinogens by enhancing the penetration of carcinogens into the cell [18]. Acetaldehyde is a metabolite of alcohol and may be the most important agent responsible for the carcinogenic effect as it is highly toxic, mutagenic, and carcinogenic [68]. A pooled analyses on alcohol reported similar risk across all areas of the large bowel [26]. However, a stronger association with alcohol for rectal cancer compared with colon cancer as seen in our paper could possibly be related to a higher degree of epithelial hyperregeneration in rectum [69]. The number of sex-specific analysis is presently too low to suggest any significant interaction by sex at the subsite level. 

There are some methodological issues in this study which may have impacted on the findings. There is considerable variability in several key characteristics of the assembled cohort studies: the follow-up time across studies included in this paper varied from 4 to 22 years, while the total number of colorectal cancer cases analyzed ranged from 126 to 2974. Short-term follow-up studies tend to accrue a lower number of cases, and the resulting estimates are subject to greater uncertainty. The substratification of cases by sex, tumor location, and exposure categories, as presented here, inevitably leads to smaller numbers and a greater degree of imprecision in the estimates, even for relatively large studies. Statistically significant associations may thus be more spurious at the subsite-specific level. However, restricting the evaluation to studies with adequate statistical power did not change the overall picture. Long-term follow-up studies will commonly accrue a greater number of cases, but are more prone to measurement error given that an increasing follow-up time raises the possibility that exposure status of the participants will change, leading to misclassification of exposure and under- or overestimation of the risk estimates. However, the natural history of colorectal cancer is on average of long duration, and exposure in the more distant past may be the most relevant when estimating subsequent risk.

Another issue is the difference in risk factor dosages between studies and that the categories compared sometimes vary considerably between studies. For instance, for calcium, in the Swedish study by Larsson et al. [47] daily intake of 1445 mg or more is compared to an intake of less than 956 mg/day, whereas in the Japanese study by Ishihara et al. [51] daily intake of 661 mg or more is compared with an intake of less than 337 mg/day. Careful reading of the exposure categories (given in the figures) is therefore necessary when evaluating the findings. In addition, the specific confounding factors adjusted for at the analysis stage differ between studies. 

Of the studies that were full-text reviewed, 76 were excluded as they did not reveal information at the level of subsite location. Given the high proportion of cohort studies failing to report subsite-specific estimates, the potential publication bias prohibited a formal meta-analysis [59]. A further rationale for this decision is the lack of uniformity in exposure categories within each risk factor. 

In summary, the strength of the association between dietary components and cancer of the large bowel may partially depend on the anatomic location within the colorectum. The most consistent finding is the stronger association between alcohol and rectal cancer, compared with alcohol and proximal and distal colon cancer. Meat (red and processed) is possibly more strongly associated with risk of distal colon cancer and rectal cancer than the risk of proximal colon cancer. For fiber, milk, and calcium there seem to be only minor differences in relative risk across the subsites. However, caution is required as the number of papers presenting risk estimates by colorectal subsite is limited, particularly for milk and garlic. Also, most of the subsite-specific analyses report non-significant findings. 

## Figures and Tables

**Table 1:** Search results as of October 8, 2012.

	Queries	Result
1	Search *colorectal neoplasms *	148374
2	Search *risk factors *	745969
3	Search *diet *	332911
4	Search *nutrition *	259739
5	Search *alcohol *	718622
6	Search *cohort study *	1253216
7	Search 3 OR 4 OR 5	1215492
8	Search 2 AND 7	81363
9	Search 1 AND 8	2099
10	Search 6 AND 9	627
11	Search *case control study *	622385
12	Search *review *	2082039
13	Search 10 NOT 11 NOT 12	356
14	Search 13 Limits: Human, English	341

**Table 2:** Characteristics of cohort studies included in the review on subsite specific dietary risk factors for colorectal cancer.

Exposure	Reference	Cohort	Cases	Sex	Age	Follow-up, yr	Adjustments***	Study Cohort, Country or Continent	Abbreviation
Red meat	Shin et al., [36]	869725 M	M: 3051 CRC, 536 PC, 751 DC, 1535 RC	B	30–80	7	1	Korean National Health System, Korea	KNHS
		395501 F	F: 1093 CRC, 236 PC, 225 DC, 451 RC						
	Cross et al., [35]	300948	2719 CRC, 1150 PC, 787 DC, 724 RC	B	50–71	7.2	2, 3, 5, 6, 7, 12	NIH-AARP Diet and Health Study, U.S.	NIH-AARP
	Sato et al., [34]	41835	396/368 CRC, 123/115 PC, 85/75 DC, 159/155 RC^∗$^	B	40–64	11	1, 2, 3, 4, 5, 6, 7, 8, 11, 12	The Miyagi Cohort Study, Japan	MCS
	Chao et al., [31]	148610	1667 CRC, 667 PC, 408 DC, 470 RC	B	50–74	8-9	1, 2, 3, 4, 5, 6, 7, 8, 10, 12, 13	The Cancer Prevention Study II Nutrition Cohort, U.S.	CPS-II
	Larsson et al., [32]	61433	733 CRC, 234 PC, 155 DC, 230 RC	F	40–75	13.9	1, 3, 6, 7, 8, 12	The Swedish Mammography Cohort, Sweden	SMC
	Norat et al., [33]	478040	1329 CRC, 351 PC, 391 DC, 474 RC	B	35–70	4.8	1, 2, 3, 4, 5, 6, 7, 8, 14	The European Prospective Investigation into Cancer and Nutrition, Europe	EPIC
	Giovannucci et al., [30]	47949	251 CRC, 69 PC, 89 DC, 46 RC	M	40–75	6	1, 3, 4, 5, 8, 9, 10, 11, 14	The Health Professionals Follow-up Study, U.S.	HPFS

Processed meat	Cross et al., [35]	300948	2719 CRC, 1150 PC, 787 DC, 724 RC	B	50–71	7.2	2, 3, 5, 6, 7, 12	NIH-AARP Diet and Health Study, U.S.	NIH-AARP
	Sato et al., [34]	41835	474 CRC, 142 PC, 100 DC, 198 RC*	B	40–64	11	1, 2, 3, 4, 5, 6, 7, 8, 11, 12	The Miyagi Cohort Study, Japan	MCS
	Chao et al., [31]	148610	1667 CRC, 667 PC, 408 DC, 470 RC	B	50–74	8-9	1, 2, 3, 4, 5, 6, 7, 8, 10, 12, 13	The Cancer Prevention Study II Nutrition Cohort, U.S.	CPS-II
	Larsson et al., [32]	61433	733 CRC, 234 PC, 155 DC, 230 RC	F	40–75	13.9	1, 3, 6, 7, 8, 12	The Swedish Mammography Cohort, Sweden	SMC
	Norat et al., [33]	478040	1329 CRC, 351 PC, 391 DC, 474 RC	B	35–70	4.8	1, 2, 3, 4, 5, 6, 7, 8, 14	The European Prospective Investigation into Cancer and Nutrition, Europe	EPIC

Fiber/ whole grain	Murphy et al., [44]	142250 M 335062 F	4517 CRC, 1298 PC, 1266 DC, 1648 RC	B	25–70	11	1, 2, 3, 4, 5, 6, 7, 8, 12, 13, 14	The European Prospective Investigation into Cancer and Nutrition, Europe	EPIC
	Egeberg et al., [43]	26630 M	M: 244 CC, 89 PC, 140 DC, 169 RC	B	50–64	10.6	1, 3, 4, 6, 8, 12, 13	The Danish Diet, Cancer and Health Prospective Cohort study, Denmark	DDCHPC
		29189 F	F: 217 CC, 84 PC, 118 DC, 114 RC						
	Kabat et al., [42]	158800	1476 CRC, 798 PC, 351 DC, 303 RC	F	50–79	7.8	1, 3, 4, 5, 6, 7, 11, 12, 13, 14	The Women's Health Initiative, U.S.	WHI
	Nomura et al., [40]	85903 M	M: 1138 CRC, 382 PC, 327 DC, 276 RC	B	45–75	7.3	1, 3, 4, 5, 6, 8, 9, 10, 11, 13, 14	The Multiethnic Cohort Study, Hawaii and Los Angeles, U.S.	MEC
		105108 F	F: 972 CRC, 356 PC, 234 DC, 179 RC						
	Schatzkin et al., [41]	489611	2974 CRC, 1139 PC, 914 DC, 858 RC*	B	50–71	5	2, 3, 4, 5, 6, 7, 8, 11, 12, 13, 14	NIH-AARP Diet and Health Study, U.S.	NIH-AARP
	Larsson et al., [39]	61433	805 CRC, 249 PC, 170 DC, 252 RC	F	40–76	14.8	1, 3, 6, 7, 12	The Swedish Mammography Cohort, Sweden	SMC
	Fuchs et al., [38]	88757	787 CRC, 281 PC, 255 DC, 143 RC*	F	34–59	16	1, 3, 4, 5, 6, 7, 8, 9, 10, 11	The Nurses' Health Study, U.S.	NHS
	Steinmetz et al., [37]	41837	212 CC, 86 PC, 120 DC*	F	55–69	5	1, 7	The Iowa Women's Health Study, U.S.	IWHS

Garlic	Steinmetz et al., [37]	41837	212 CC, 86 PC, 120 DC*	F	55–69	5	1, 7	The Iowa Women's Health Study, U.S.	IWHS

Milk	Larsson et al., [47]	45306	449 CRC, 124 PC, 131 DC, 173 RC	M	45–79	6.7	1, 3, 4, 5, 6, 7, 8, 10, 11, 12, 14	The Cohort of Swedish Men, Sweden	COSM
	Larsson et al., [46]	60708	798 CRC, 246 PC, 170 DC, 249 RC	F	40–76	14.8	1, 3, 6, 7, 12	The Swedish Mammography Cohort, Sweden	SMC
	McCullough et al., [45]	60866	421 CRC, 124 PC, 103 DC, 119 RC	M	50–74	4-5	1, 3, 4, 5, 6, 7, 11, 12	The Cancer Prevention Study II Nutrition Cohort, U.S.	CPS-II

Calcium	Ishihara et al., [51]	35194	464 CRC, 129 PC, 183 DC, 146 RC*	M	45–74	7.8	1, 3, 4, 5, 6, 7, 8, 14	The Japan Public Health Center-based Prospective Study, Japan	JPHC
	Larsson et al., [47]	45306	449 CRC, 124 PC, 131 DC, 173 RC	M	45–79	6.7	1, 3, 4, 5, 6, 7, 8, 10, 11, 12, 14	The Cohort of Swedish Men, Sweden	COSM
	Flood et al., [50]	45354	482 CRC, 172 PC, 112 DC, 74 RC	F	61.9 (mean)	8.5	1, 7	The Breast Cancer Detection Demonstration Project, U.S.	BCDDP
	McCullough et al., [45]	60866	421 CRC, 124 PC, 103 DC, 119 RC	M	50–74	4-5	1, 3, 4, 5, 6, 7, 11, 12	The Cancer Prevention Study II Nutrition Cohort, U.S.	CPS-II
	Terry et al., [48]	61463	572 CRC, 164 PC, 121 DC, 191 RC	F	39–75	11.3	1, 3, 6, 7, 8, 12	The Swedish Mammography Cohort, Sweden	SMC
	Wu et al., [49]	87998 F 47344 M	1025 CC, 426 PC, 411 DC	B	30–55 F 40–75 M	16 10	1, 3, 4, 5, 6, 8, 10, 11, 13, 14	The Nurses' Health Study, U.S. and The Health Professionals Follow-up Study, U.S.	NHS & HPFS
	Stemmermann et al., [28]	8006	277 CRC, 43 PC, 33 TDC, 113 SC, 88 RC	M	46–68	19–22	1	Japan Hawaii Cancer Study, Japan	JHCS

Alcohol	Razzak et al., [29]	41836	1255 CRC, 633 PC, 594 DCR	F	55–69	12	1, 3, 4, 5, 6, 7, 13	The Iowa Women's Health study, U.S.	IWHS
	Shin et al., [36]	869725 M	M: 3051 CRC, 536 PC, 751 DC, 1535 RC	B	30–80	7	1	Korean National Health System, Korea	KNHS
		395501 F	F: 1093 CRC, 236 PC, 225 DC, 451 RC						
	Park et al., [58]	153000	M: 241 CRC, 78 PC, 65 DC, 81 RC F: 217 CRC, 76 PC, 54 DC, 69 RC	B	26–84		1, 3, 4, 5, 6, 7, 12, 14	UK Dietary Cohort Consortium, UK	UKDCC
	Bongaerts et al., [57]	4118**	M: 881 CRC, 240 PC, 293 DC, 232 RC F: 622 CRC, 254 PC, 189 DC, 108 RC	B	55–69	13.3	1, 2, 3, 4, 6, 7, 11	The Netherlands Cohort study, The Netherlands	NLCS
	Akhter et al., [55]	21199	307 CRC, 78 PC, 78 DC, 131 RC	M	40–64	11	1, 3, 4, 5, 6, 11, 12	The Miyagi Cohort study, Japan	MCS
	Ferrari et al., [56]	478732	1833 CRC, 476 PC, 528 DC, 649 RC	B	35–70	6.2	1, 2, 3, 4, 5, 7, 12	The European Prospective Investigation into Cancer and Nutrition, Europe	EPIC
	Pedersen et al., [54]	29132	613 CRC, 159 PC, 202 DC, 202 RC	B	23–95	14.7	1, 2, 5, 14	The Copenhagen Centre for Prospective Population Studies, Denmark	CCPPS
	Giovannucci et al., [53]	47931	205 CC, 69 PC, 89 DC, 46 RC	M	40–75	6	1, 3, 4, 5, 6, 7, 9, 10, 11, 14	The Health Professionals Follow-up Study, U.S.	HPFS
	Klatsky et al., [27]	106203	203 CC, 69 PC, 52 TDC, 77 SC, 66 RC	B		7	1, 2, 5, 6, 12, 14	Northern California Kaiser Permanente Study, U.S.	NCKPS
	Wu et al., [52]	11644	M: 58 CRC F: 68 CRC	B		4.5	1	Retirement Community of Los Angeles, U.S.	RCLA

M: male, F: female, B: both sexes, CRC: colorectal cancer, PC: proximal colon cancer, DC: distal colon cancer, RC: rectal cancer, DCR: distal colorectal cancer, TDC: transverse and descending colon cancer, SC: sigmoid colon cancer. *The numbers come, all or partly, from tables, and may thus not include all cases in the cohort. **Subcohort. ***The risk estimates are adjusted for: 1: age, 2: sex, 3: BMI, 4: physical activity, 5: smoking, 6: dietary items, 7: energy intake, 8: alcohol intake, 9: history of polyps, 10: NSAIDS/aspirin, 11: family history of CRC, 12: education, 13: hormone replacement therapy or oral contraceptive use, 14: others. ^$^The numbers vary according to the exposure analyzed.
